# Positive Selection Inhibits Plasmid Coexistence in Bacterial Genomes

**DOI:** 10.1128/mBio.00558-21

**Published:** 2021-05-11

**Authors:** Laura Carrilero, Anastasia Kottara, David Guymer, Ellie Harrison, James P. J. Hall, Michael A. Brockhurst

**Affiliations:** a Division of Evolution and Genomic Sciences, School of Biological Sciences, University of Manchester, Manchester, United Kingdom; b Department of Animal and Plant Sciences, University of Sheffield, Sheffield, United Kingdom; c Department of Biology, University of York, York, United Kingdom; d Department of Evolution, Ecology and Behaviour, Institute of Infection, Veterinary and Ecological Sciences, University of Liverpool, Liverpool, United Kingdom; University of Toronto

**Keywords:** experimental evolution, horizontal gene transfer, plasmid biology

## Abstract

Plasmids play an important role in bacterial evolution by transferring niche-adaptive functional genes between lineages, thus driving genomic diversification. Bacterial genomes commonly contain multiple, coexisting plasmid replicons, which could fuel adaptation by increasing the range of gene functions available to selection and allowing their recombination. However, plasmid coexistence is difficult to explain because the acquisition of plasmids typically incurs high fitness costs for the host cell. Here, we show that plasmid coexistence was stably maintained without positive selection for plasmid-borne gene functions and was associated with compensatory evolution to reduce fitness costs. In contrast, with positive selection, plasmid coexistence was unstable despite compensatory evolution. Positive selection discriminated between differential fitness benefits of functionally redundant plasmid replicons, retaining only the more beneficial plasmid. These data suggest that while the efficiency of negative selection against plasmid fitness costs declines over time due to compensatory evolution, positive selection to maximize plasmid-derived fitness benefits remains efficient. Our findings help to explain the forces structuring bacterial genomes: coexistence of multiple plasmids in a genome is likely to require either rare positive selection in nature or nonredundancy of accessory gene functions among the coexisting plasmids.

## INTRODUCTION

Plasmids play an important role in the evolution of bacterial genomes, promoting evolutionary divergence by transferring niche-adaptive accessory gene functions between lineages (1–3). Bioinformatic analyses suggest that carriage by single bacterial host cells of multiple, coexisting plasmid replicons (also termed plasmid coinfection) is more commonly observed than would be expected by chance (4). Accumulation of multiple, coexisting plasmid replicons thus drives genome expansion and can lead to the evolution of multipartite genomes (5–7). Moreover, this process could potentially fuel evolutionary innovation through reassortment of new combinations of accessory functions and the formation of new mobile genetic elements through plasmid-plasmid recombination (8–10). Nevertheless, the observed high rates of plasmid coinfection are surprising, given that plasmid acquisition usually disrupts normal cellular function and is often associated with large fitness costs for the host cell (11–15).

One explanation for abundant plasmid coexistence in bacterial genomes is that the fitness costs of acquiring multiple plasmids could be less than additive. Positive epistasis between plasmid costs could permit the accumulation of multiple plasmids by reducing the cost for plasmid bearers of acquiring additional plasmids (4, 16, 17); however, positive epistatic interactions among plasmid costs are not universal (4). Moreover, as we show in this study, the methods by which positive epistasis has been previously estimated (i.e., competition of plasmid carriers against plasmid-free cells) (4) may not measure the actual cost of plasmid coinfection. Compensatory evolution to ameliorate the cost of plasmids has been described for a wide range of plasmid systems and prevents plasmid loss by weakening negative selection against the plasmid backbone over time (18–22). If the fitness costs of multiple plasmids have common mechanistic causes, it is possible that the same compensatory mechanisms could simultaneously ameliorate the costs of multiple plasmids, which may in turn promote stable plasmid coinfection (16, 20). The role of positive selection for beneficial plasmid-encoded accessory gene functions in plasmid coinfection is less well studied. Positive selection could promote plasmid coexistence if the fitness benefits of carrying multiple plasmids outweighed the accumulative fitness costs, or, alternatively, may inhibit coexistence by selecting for consolidation of beneficial functions onto fewer replicons, accompanied by the loss of a redundant, costly plasmid backbone(s) (14, 15). Understanding the roles of these various mechanisms in plasmid coexistence requires experimental tests, but while the fitness costs of single infection and coinfection by plasmids have been estimated, studies tracking the longer-term dynamics of plasmid coexistence in bacterial populations are lacking.

Here, we considered the experimental coexistence dynamics for two distantly related, naturally co-occurring conjugative mercury resistance plasmids—pQBR103 and pQBR57—originally isolated from a field site in the United Kingdom (23, 24). Each of the plasmids individually causes a substantial fitness cost in the host bacterium Pseudomonas fluorescens SBW25 (25). Both plasmids encode nearly identical copies of the *mer* mercury resistance operon on transposon Tn*5042* (25, 26), which allows plasmid bearers to reduce toxic Hg(II) to Hg, providing a fitness benefit to plasmid-carriers at increased Hg(II) concentrations (25). Although SBW25(pQBR57) outcompetes SBW25(pQBR103) in the absence of mercury, this competitive hierarchy is reversed in mercury-containing environments (25). This suggests that while pQBR57 imposes a lower fitness cost, it also provides a lesser fitness benefit in the presence of mercury, relative to pQBR103 in singly infected SBW25 cells. Both plasmids are maintained individually in bacterial populations, and while this appears in each case to be linked to compensatory evolution (19, 27), unlike pQBR103, pQBR57 is capable of a high rate of conjugative transfer, which contributes to its survival and spread, particularly in the absence of mercury (28, 29). Multiple mechanisms of compensatory evolution have been described previously for the pQBR plasmids. Specifically, chromosomal compensatory mutations that occur either in *gacA*-*gacS*, encoding a two-component global regulatory system, or in *PFLU4242*, encoding a hypothetical protein with two domains of unknown function, reduce the cost of these and other pQBR plasmids individually (18, 19).

Replicate laboratory populations of SBW25 cells that were originally either singly infected or coinfected with the plasmids pQBR103 and pQBR57 were propagated by serial transfer with or without positive selection [i.e., addition of mercury(II) chloride] for approximately ∼265 bacterial generations. We tracked bacterial population densities and the dynamics of mercury resistance over time and used multiplex PCR to determine the plasmid carriage status of mercury-resistant clones. We show that plasmid coexistence was stable in populations without positive selection, whereas positive selection drove the loss of pQBR57 and the dominance of SBW25 carrying only pQBR103. Loss of plasmid coexistence occurred despite compensatory evolution to ameliorate plasmid fitness costs and was caused by positive selection discriminating between the differential fitness benefits of the plasmids and retaining only the more beneficial plasmid.

## RESULTS

### Temporal dynamics of mercury resistance and plasmid carriage.

**FIG 1:**
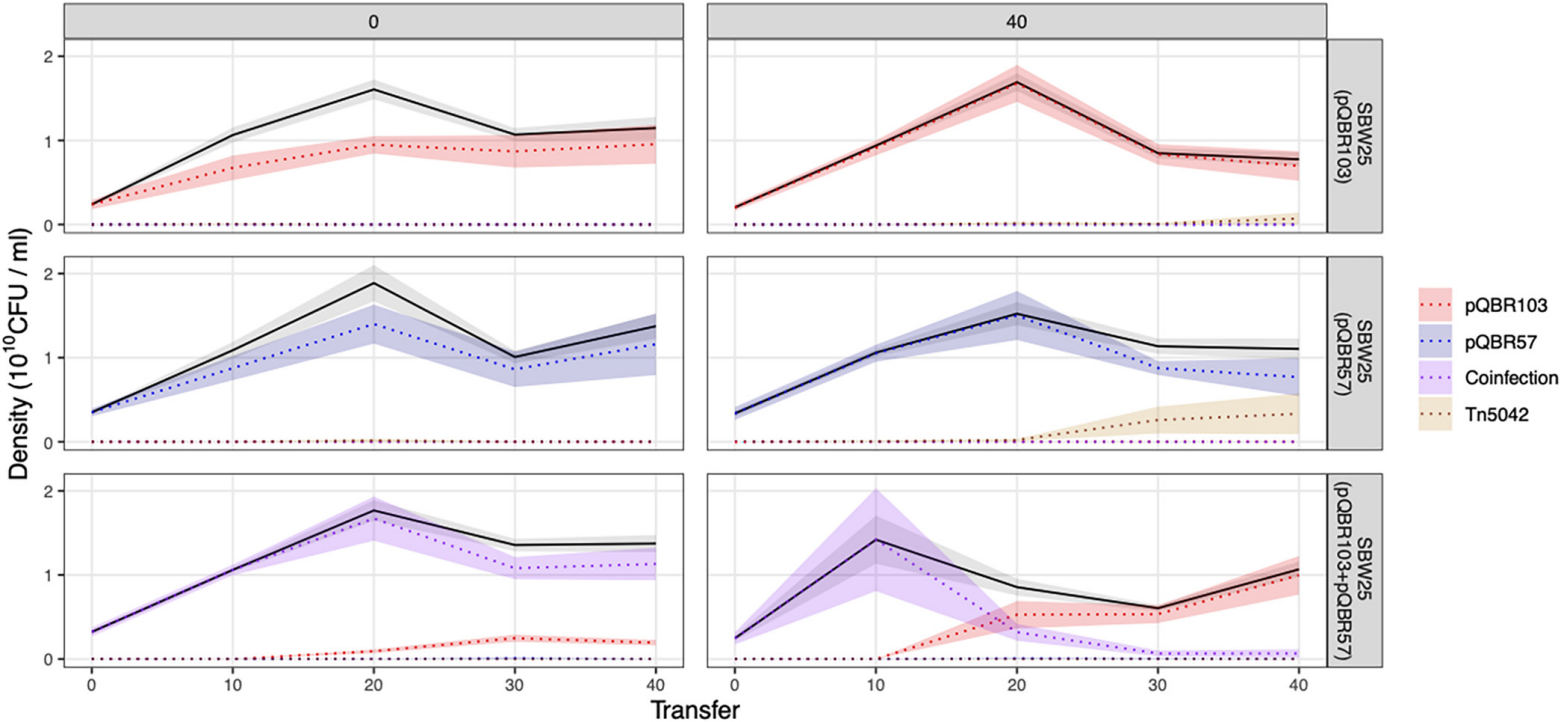
Population and mercury resistance genotype dynamics over time. Plots are faceted by treatment, horizontally by mercury treatment [0 μM or 40 μM Hg(II) chloride] and vertically by plasmid treatment (pQBR103 only, pQBR57 only, or both pQBR103 and pQBR57). Solid black lines show means (*n* = 6), and gray shaded areas show standard errors for the total bacterial population density estimated from colony counts. Colored dotted lines and correspondingly colored shaded areas show the means and standard errors for the population density of pQBR103-only carriers (red), pQBR57-only carriers (blue), pQBR103+pQBR57 carriers (purple), and chromosomal Tn*5042*-carrying plasmid-free cells (gold) inferred from their genotype frequencies. Plots for the population and genotype dynamics of individual replicates are shown in Fig. S1. Raw data are provided in Data Set S1.

To study the effect of positive selection on the dynamics of plasmid coexistence, we propagated replicate populations of SBW25 carrying either both plasmids, pQBR103 alone, or pQBR57 alone, both with and without selective levels of Hg(II) chloride, by serial transfer for approximately 265 bacterial generations. We also propagated replicate plasmid-free control SBW25 populations without Hg(II) chloride. Mercury resistance (Hg^r^) was maintained near fixation under positive selection for all plasmid treatments, but without positive selection, its frequency varied according to plasmid treatment (Fig. 1) [comparison of cumulative Hg^r^ frequency between plasmid treatments without Hg(II) selection: Welch’s analysis of variance (ANOVA); *W*_2,6.685_ = 18.32, *P* = 0.0019]. Without positive selection, whereas Hg^r^ remained at high frequency in the two-plasmid treatment, Hg^r^ declined in the single-plasmid treatments and significantly so in the pQBR103-only treatment [Dunnett’s T3 test for pairwise comparisons of plasmid treatments without Hg(II) selection: for pQBR103 versus both, *P* = 0.0044; for pQBR57 versus both, *P* = 0.4038; for pQBR103 versus pQBR57 only, *P* = 0.3157). The Hg^r^ phenotype indicates the presence of the Tn*5042*-borne *mer* operon within the cell, which could be explained by the maintenance of one or both plasmids or by the relocation of the Tn*5042* to the chromosome accompanied by plasmid loss (19, 28, 30). We therefore used multiplex PCR to determine the presence of the Tn*5042* and of each plasmid. In the single-plasmid treatments, whereas plasmid-encoded Hg^r^ predominated in populations propagated without positive selection, we observed the invasion of plasmid-free cells carrying a chromosomal Tn*5042* in some replicates with positive selection (Fig. 1; Fig. S1) (2/6 replicates of the pQBR57-only treatment; 1/6 replicates of the pQBR103-only treatment). Plasmid dynamics also varied with positive selection in the two-plasmid treatments. Plasmid coexistence within cells was maintained at higher frequency in populations without positive selection than in those propagated with positive selection (Fig. 1) (comparison of cumulative coinfection frequency: Mann-Whitney U test, U = 0, *P* = 0.0022). This was driven by the loss of pQBR57 from initially coinfected cells under positive selection, such that plasmid-coinfected cells were replaced by cells carrying pQBR103-only in 4 of 6 replicates (Fig. S1). Taken together, these data suggest that positive selection inhibited plasmid coexistence and, furthermore, that the pQBR57 backbone was lost more readily than the pQBR103 backbone under positive selection for mercury resistance.

10.1128/mBio.00558-21.1 FIG S1 Population dynamics and mercury resistance genotype dynamics over time per replicate. Plots are faceted by treatment and replicate, horizontally by mercury treatment [0 μM or 40 μM Hg(II) chloride], and then by replicate, vertically by plasmid treatment (pQBR103 only, pQBR57 only, or both pQBR103 and pQBR57). Solid black lines show the bacterial population density estimated from colony counts. Colored shaded areas show the population density of pQBR103-only carriers (red), pQBR57-only carriers (blue), coinfected pQBR103+pQBR57 carriers (purple), and chromosomal-Tn*5042*-carrying plasmid-free cells (grey) inferred from their genotype frequencies. Download FIG S1, JPG file, 0.4 MB. Copyright © 2021 Carrilero et al. 2021 Carrilero et al. https://creativecommons.org/licenses/by/4.0/ This content is distributed under the terms of the Creative Commons Attribution 4.0 International license.

10.1128/mBio.00558-21.10 DATA SET S1 Experimental raw data and table of called sequence variants. Download Data Set S1, XLSX file, 0.09 MB. Copyright © 2021 Carrilero et al. 2021 Carrilero et al. https://creativecommons.org/licenses/by/4.0/ This content is distributed under the terms of the Creative Commons Attribution 4.0 International license.

### Fitness effects of plasmid carriage.

**FIG 2:**
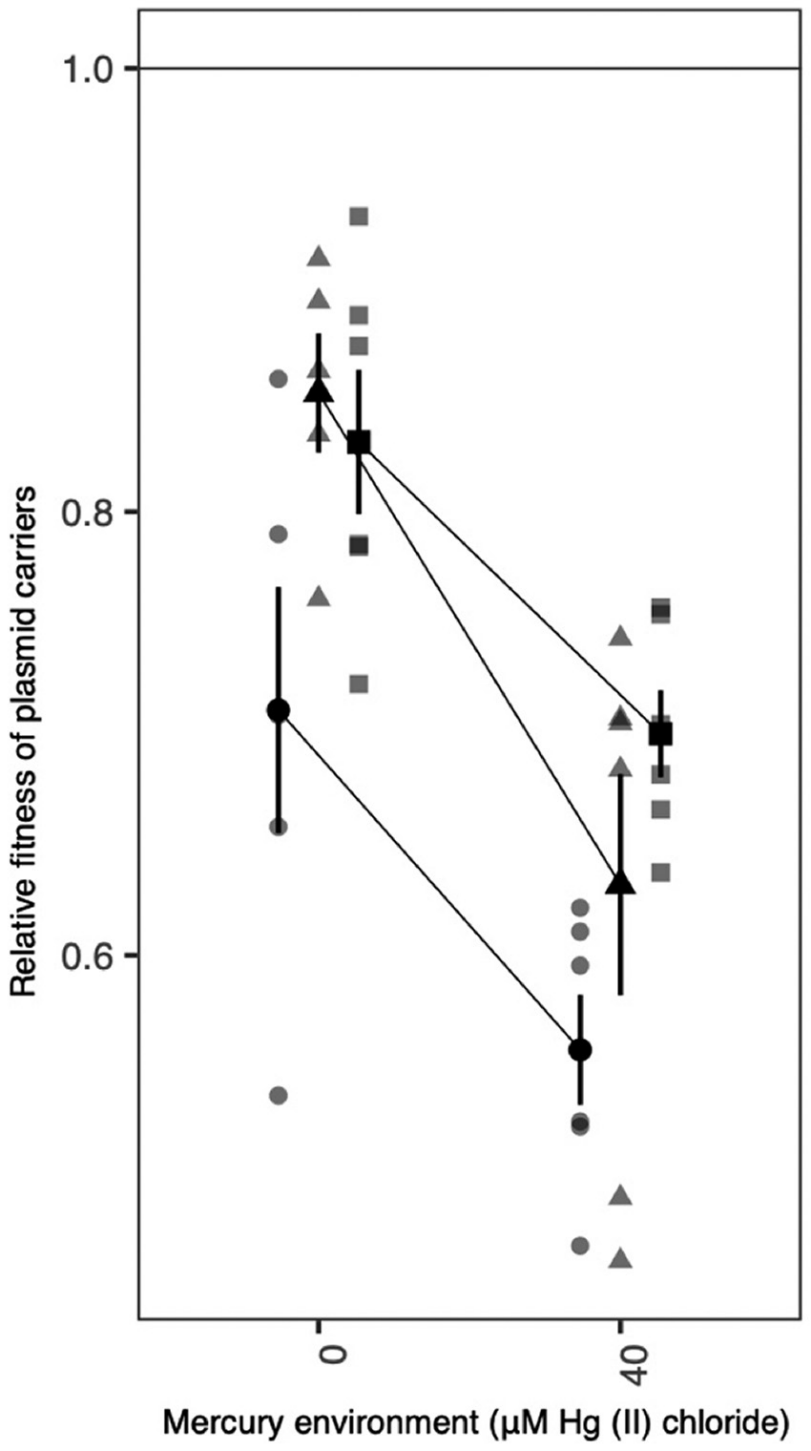
Fitness of plasmid carriers relative to SBW25(Tn*5042*) competed with or without Hg(II) chloride. Black symbols show means and standard errors for relative fitness of SBW25(pQBR103) (circles), SBW25(pQBR57) (triangles), and SBW25(pQBR103+pQBR57) (squares) against SBW25(Tn*5042*). Gray symbols in corresponding shapes show the individual replicate values (*n* = 5 or 6). All competition assays were 6-fold replicated except for SBW25(pQBR103) versus SBW25(Tn*5042*) in 0 μM Hg(II) chloride and SBW25(pQBR57) versus SBW25(Tn*5042*) in 0 μM Hg(II) chloride, where one replicate each was lost due to contamination. Raw data are provided in Data Set S1.

To understand the effect of positive selection on the relative fitness of the various Hg^r^ genotypes observed here—i.e., chromosomal Tn*5042*, either plasmid alone, or both plasmids together—we performed a series of competition experiments. First, we tested how positive selection affected the cost of carrying the plasmid backbone(s) by competing SBW25 plasmid bearers against an isogenic ancestral SBW25 carrying a chromosomal copy of Tn*5042* [SBW25(Tn*5042*)]. Here, in each pairwise competition, both competitors are resistant to mercury, but the plasmid bearers must pay the additional fitness costs of maintaining the plasmid backbone(s). Plasmid bearers varied in fitness relative to SBW25(Tn*5042*) according to their plasmid complement: pQBR103 had a higher fitness cost than either pQBR57 or both plasmids together (Fig. 2) (ANOVA: plasmid main effect, *F*_2,28_ = 7.813, *P* = 0.002; Tukey pairwise contrasts: pQBR103 versus pQBR57, *P* = 0.0217; pQBR103 versus both, *P* = 0.0019; pQBR57 versus both, *P* = 0.6399). Moreover, the addition of Hg(II) chloride increased the fitness cost of plasmid carriage (mercury main effect, *F*_1,28_ = 31.298; *P* = 5.48 × 10^−6^), suggesting that once the benefit of mercury resistance is negated, Hg(II) chloride increases the costs of the plasmid backbones *per se*. Together, these data confirm previous studies reporting a higher individual cost of the pQBR103 backbone relative to the pQBR57 backbone alone (25) and, moreover, explain the loss of redundant plasmid replicons under positive selection seen here and in other studies (28, 31).

**FIG 3:**
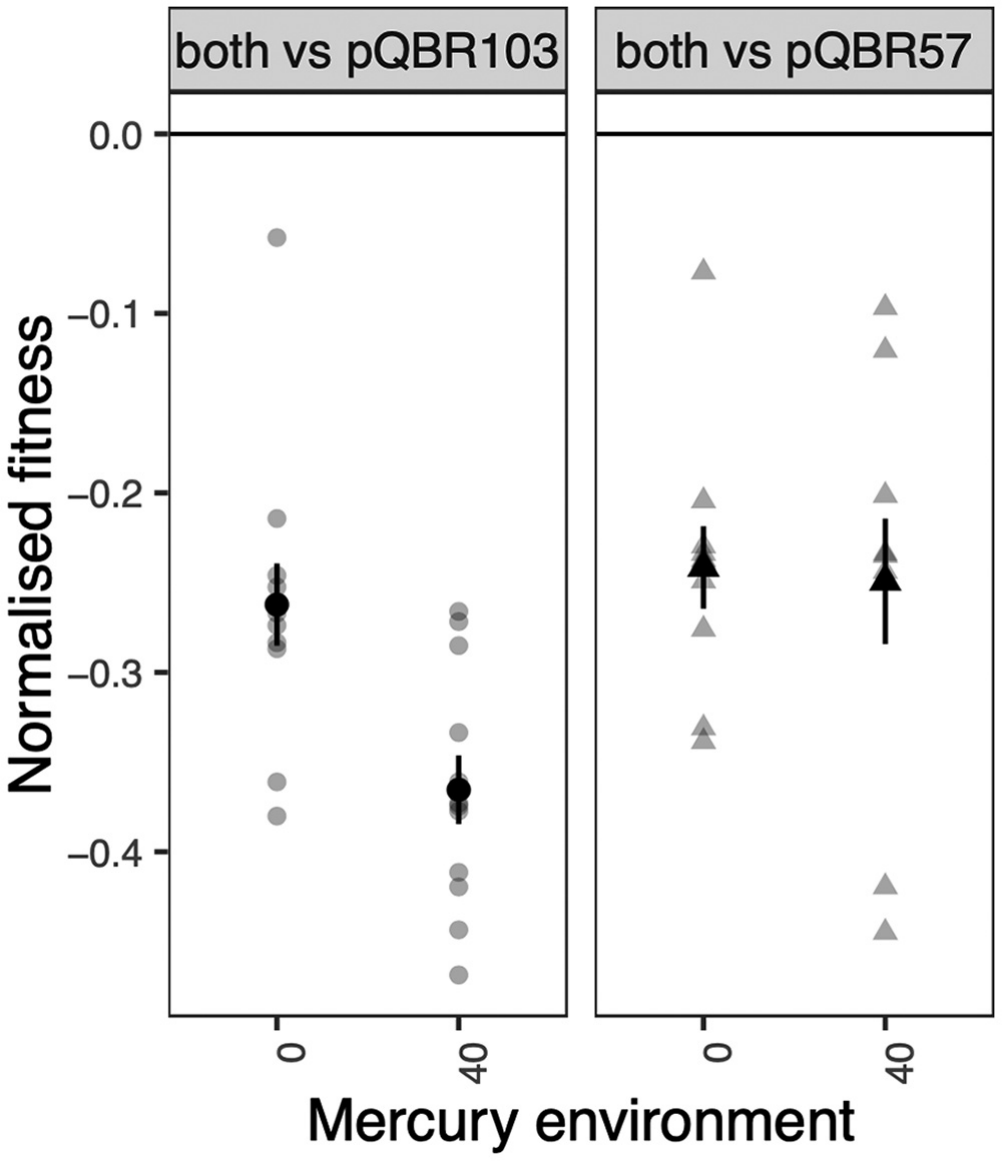
Fitness of coinfected plasmid carriers against singly infected plasmid carriers competed with or without Hg(II) chloride. Black symbols show means and standard errors for normalized fitness of SBW25(pQBR103+pQBR57) competing against SBW25(pQBR103) (circles; left) or SBW25(pQBR57) (triangles; right). Gray symbols in corresponding shapes show the individual replicate values (*n* = 10 or 12). Competition assays against SBW25(pQBR103) were 12-fold replicated, whereas competition assays against SBW25(pQBR57) were 10-fold replicated due to the loss of replicates to contamination. Raw data are provided in Data Set S1.

Nevertheless, these data cannot explain the preferential loss of the pQBR57 backbone from coinfected cells that we observed. To further explore this, we next directly competed SBW25 coinfected with both plasmids against SBW25 carrying either of the plasmids alone, both with and without positive selection. We found that the relative fitness of coinfected SBW25 was lower when it was competed against SBW25(pQBR103) than against SBW25(pQBR57) (Fig. 3) (ANOVA; plasmid main effect, *F*_1,40_ = 7.438; *P* = 0.009435) and was reduced by the presence of Hg(II) in the media (mercury main effect, *F*_1,40_ = 5.727; *P* = 0.02149), while the interaction of these factors was marginally nonsignificant (plasmid-mercury interaction, *F*_1,40_ = 3.616; *P* = 0.06443). *Post hoc* pairwise comparisons were consistent with positive selection favoring the loss of pQBR57 from coinfected cells, as we observed in our serial transfer experiment: The addition of Hg(II) chloride further reduced the fitness of coinfected SBW25(pQBR57+pQBR103) when it was competed against SBW25(pQBR103) [Tukey pairwise comparison; with versus without Hg(II) chloride, *P* = 0.0203] but not when it was competed against SBW25(pQBR57) [Tukey pairwise comparison; with versus without Hg(II) chloride, *P* = 0.9967], such that the fitness of coinfected cells was higher in competitions against SBW25(pQBR57) than against SBW25(pQBR103) in the presence of Hg(II) chloride (Tukey pairwise comparison; *P* = 0.0113). Taken together and considered along with previous findings (25), these data suggest that although the pQBR103 backbone is costlier than the pQBR57 backbone when carried alone, pQBR103 had a higher net benefit in mercury-containing environments than pQBR57. Thus, the preferential loss of pQBR57 from coinfected cells under positive selection is better explained by selection favoring the more beneficial plasmid rather than selection against plasmid costs.

### Compensatory mutations and their fitness effects.

**FIG 4:**
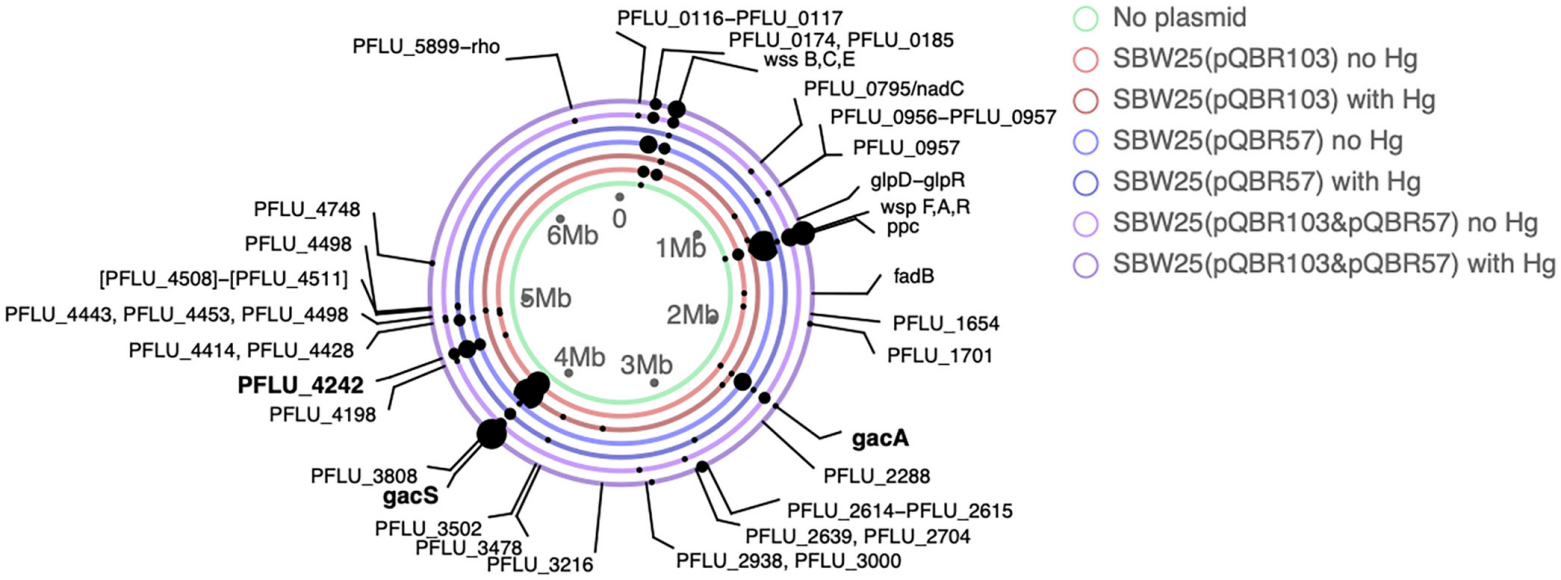
Parallel evolution plot summarizing the mutations arising in evolved clones per treatment. Rings represent the SBW25 chromosome plotted separately for each treatment as indicated by the color of the ring (see the legend). Dots indicate genetic loci where mutations (excluding hypermutator clones) were observed in that treatment; the size of the dot corresponds to the number of replicate populations in which a mutation at that locus occurred per treatment. Loci previously associated with compensatory mutations are highlighted in bold. In populations where more than one plasmid-bearing clone was sequenced, only mutations present in the dominant genotype are shown here. Hypermutator clones are not shown to avoid overplotting. Plots for all sequenced clones are provided in Fig. S2 to S8. The full table of called sequence variants is provided in Data Set S1.

**FIG 5:**
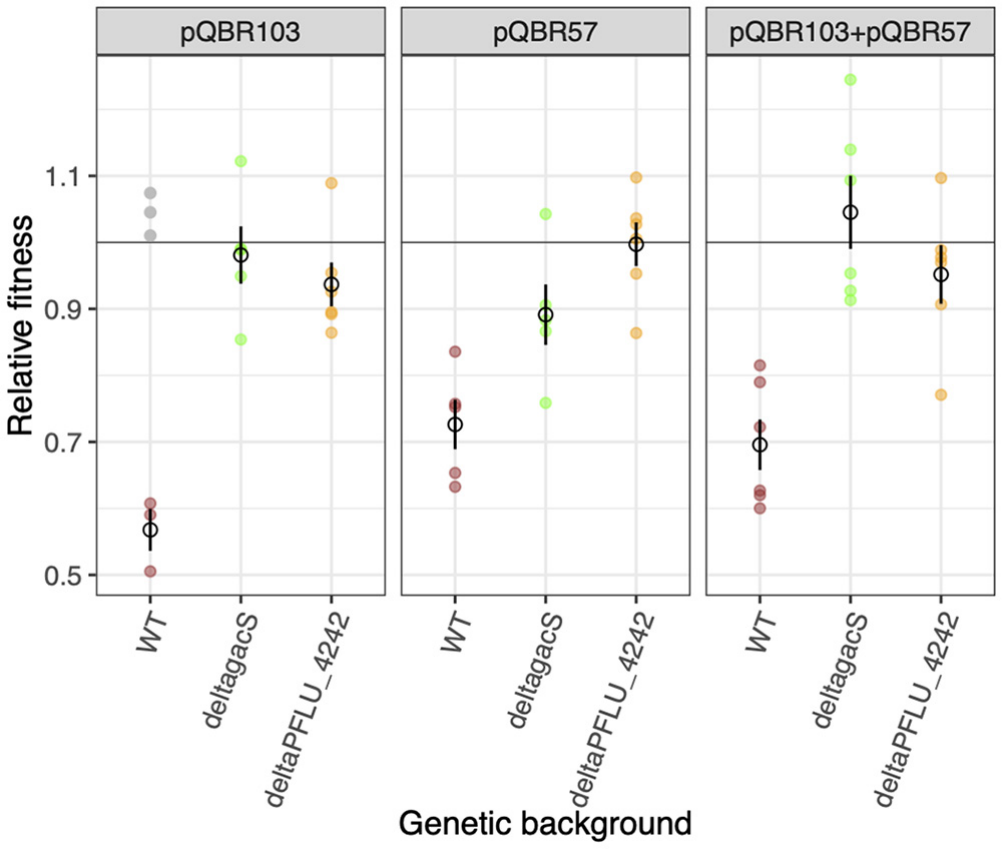
Compensatory mutations ameliorate the fitness costs of plasmid carriage. Plots are faceted horizontally by the plasmid status of the plasmid-carrying competitor. Open symbols show means (*n* = 6) and standard errors of fitness for plasmid-carrying strains relative to plasmid-free SBW25. Colored filled dots denote individual replicate values for SBW25 (maroon), SBW25-ΔgacS (green), or SBW25-ΔPFLU4242 (orange) genotypes. Outliers (gray) were excluded from the statistical analysis. Raw data are provided in Data Set S1.

A potential alternative explanation for the contrasting patterns of plasmid maintenance among treatments would be differences in the propensity for compensatory evolution to occur according to plasmid content or positive selection. To test this, we obtained the whole-genome sequences of one randomly chosen evolved clone per Hg^r^ genotype present at a frequency greater than 10% per population at the end of the serial transfer experiment. There was no evidence for differential compensatory evolution according to treatment: all sequenced plasmid-bearing evolved clones carried a mutation in a known compensatory locus. Specifically, we observed mutations in *gacA*, *gacS*, or *PFLU4242* or in regions immediately upstream of compensatory loci (Fig. 4; data for all sequenced clones are provided in Fig. S2 to S8). In contrast, mutations at these loci were never observed in evolved clones from the plasmid-free control populations. To confirm that loss of either *gacS* or *PFLU4242* was sufficient to ameliorate the cost of plasmid coinfection, we compared the effect of compensatory mutations on the fitness of plasmid bearers carrying either one or both plasmids relative to the plasmid-free ancestor using competition experiments. Consistent with a role for these genes in compensatory evolution, deletion of either *gacS* or *PFLU4242* reduced the fitness cost of carrying plasmids (Fig. 5). However, while deletion of either gene completely ameliorated the cost of carrying either both plasmids or pQBR103 alone, only deletion of *PFLU4242* completely ameliorated the cost of carrying pQBR57 alone, whereas deletion of *gacS* offered only partial amelioration (ANOVA; strain by plasmid interaction, *F*_4, 39_ = 2.628, *P* = 0.049). Finally, we tested whether the effects of compensatory mutations on plasmid bearers varied according to the mercury environment by measuring growth kinetics of plasmid-bearing strains with either the wild-type, *gacS*, or *PFLU4242* deletion genotype in the presence or absence of Hg(II) chloride. Although Hg(II) chloride slightly reduced growth overall (Fig. S9) (ANOVA, main effect of mercury, *F*_1,52_ = 5.400, *P* = 0.024), it did not affect the growth of either of the compensatory mutants carrying either pQBR57, pQBR103, or both plasmids (pairwise contrasts between mercury environments for each genotype-plasmid combination, all *P* > 0.05), confirming that neither mechanism of compensatory evolution’s efficacy was affected by mercury. Thus, compensatory evolution occurred in all treatments and was effective at ameliorating the additional cost of carrying multiple plasmids. While this may explain the stability of plasmid coexistence without positive selection, it cannot explain the decline in plasmid coexistence due to loss of pQBR57 with positive selection, which instead appears to have been driven by the differential benefits of the plasmids in the presence of Hg(II) chloride.

10.1128/mBio.00558-21.2 FIG S2 Parallel evolution plot showing the mutations arising in evolved clones from the 0 μM Hg(II) chloride, no-plasmid treatment. Rings represent the SBW25 chromosome plotted separately for each replicate. Dots indicate genetic loci where mutations were observed in that treatment; the size of the dot corresponds to the number of mutations occurred. Loci previously associated with compensatory mutations are highlighted in bold for all clones, and the labels for the rest of the loci were plotted only for the no-hypermutator clones to avoid overplotting. When large mutations occurred, partially deleted flanking genes appear in square brackets and completely deleted flanking genes appear in curly brackets. Download FIG S2, JPG file, 0.2 MB. Copyright © 2021 Carrilero et al. 2021 Carrilero et al. https://creativecommons.org/licenses/by/4.0/ This content is distributed under the terms of the Creative Commons Attribution 4.0 International license.

10.1128/mBio.00558-21.3 FIG S3 Parallel evolution plot showing the mutations arising in evolved clones from the 0 μM Hg(II) chloride, pQBR103-only treatment. Rings represent the SBW25 chromosome plotted separately for each replicate. Dots indicate genetic loci where mutations were observed in that treatment; the size of the dot corresponds to the number of mutations that occurred. Loci previously associated with compensatory mutations are highlighted in bold for all clones, and the labels for the rest of the loci were plotted only for the no-hypermutator clones to avoid overplotting. When large mutations occurred, partially deleted flanking genes appear in square brackets and completely deleted flanking genes appear in curly brackets. Download FIG S3, JPG file, 0.3 MB. Copyright © 2021 Carrilero et al. 2021 Carrilero et al. https://creativecommons.org/licenses/by/4.0/ This content is distributed under the terms of the Creative Commons Attribution 4.0 International license.

10.1128/mBio.00558-21.4 FIG S4 Parallel evolution plot showing the mutations arising in evolved clones from the 0 μM Hg(II) chloride, pQBR57-only treatment. Rings represent the SBW25 chromosome plotted separately for each replicate. Dots indicate genetic loci where mutations were observed in that treatment; the size of the dot corresponds to the number of mutations that occurred. Loci previously associated with compensatory mutations are highlighted in bold for all clones, and the labels for the rest of the loci were plotted only for the no-hypermutator clones to avoid overplotting. When large mutations occurred, partially deleted flanking genes appear in square brackets and completely deleted flanking genes appear in curly brackets. Download FIG S4, JPG file, 0.3 MB. Copyright © 2021 Carrilero et al. 2021 Carrilero et al. https://creativecommons.org/licenses/by/4.0/ This content is distributed under the terms of the Creative Commons Attribution 4.0 International license.

10.1128/mBio.00558-21.5 FIG S5 Parallel evolution plots showing the mutations arising in evolved clones from the 0 μM Hg(II) chloride, two-plasmid treatment. (A) Evolved clones that contained both plasmids; (B) evolved clones that contained pQBR103 only. Rings represent the SBW25 chromosome plotted separately for each replicate. Dots indicate genetic loci where mutations were observed in that treatment; the size of the dot corresponds to the number of mutations that occurred. Loci previously associated with compensatory mutations are highlighted in bold for all clones, and the labels for the rest of the loci were plotted only for the no-hypermutator clones to avoid overplotting. When large mutations occurred, partially deleted flanking genes appear in square brackets and completely deleted flanking genes appear in curly brackets. Download FIG S5, JPG file, 0.3 MB. Copyright © 2021 Carrilero et al. 2021 Carrilero et al. https://creativecommons.org/licenses/by/4.0/ This content is distributed under the terms of the Creative Commons Attribution 4.0 International license.

10.1128/mBio.00558-21.6 FIG S6 Parallel evolution plots showing the mutations arising in evolved clones from the 40 μM Hg(II) chloride, pQBR103-only treatment. (A) Evolved clones that contained pQBR103; (B) evolved clone that had lost pQBR103 but retained the Tn*5042* mercury resistance transposon (replicate B). Rings represent the SBW25 chromosome plotted separately for each replicate. Dots indicate genetic loci where mutations were observed in that treatment; the size of the dot corresponds to the number of mutations occurred. Loci previously associated with compensatory mutations are highlighted in bold for all clones, and the labels for the rest of the loci were plotted only for the no-hypermutator clones to avoid overplotting. When large mutations occurred, partially deleted flanking genes appear in square brackets and completely deleted flanking genes appear in curly brackets. Download FIG S6, JPG file, 0.3 MB. Copyright © 2021 Carrilero et al. 2021 Carrilero et al. https://creativecommons.org/licenses/by/4.0/ This content is distributed under the terms of the Creative Commons Attribution 4.0 International license.

10.1128/mBio.00558-21.7 FIG S7 Parallel evolution plots showing the mutations arising in evolved clones from the 40 μM Hg(II) chloride, pQBR57 alone treatment. (A) Evolved clones that contained pQBR57; (B) evolved clones that had lost pQBR57 but retained the Tn*5042* mercury resistance transposon (replicates A and B). Rings represent the SBW25 chromosome plotted separately for each replicate. Dots indicate genetic loci where mutations were observed in that treatment; the size of the dot corresponds to the number of mutations that occurred. Loci previously associated with compensatory mutations are highlighted in bold for all clones, and the labels for the rest of the loci were plotted only for the no-hypermutator clones to avoid overplotting. When large mutations occurred, partially deleted flanking genes appear in square brackets and completely deleted flanking genes appear in curly brackets. Download FIG S7, JPG file, 0.2 MB. Copyright © 2021 Carrilero et al. 2021 Carrilero et al. https://creativecommons.org/licenses/by/4.0/ This content is distributed under the terms of the Creative Commons Attribution 4.0 International license.

10.1128/mBio.00558-21.8 FIG S8 Parallel evolution plots showing the mutations arising in evolved clones from the 40 μM Hg(II) chloride, two-plasmid treatment. (A) Evolved clones that contained both pQBR57 and pQBR103 (replicates B and C); (B) evolved clones that had lost pQBR57 but retained pQBR103. Rings represent the SBW25 chromosome plotted separately for each replicate. Dots indicate genetic loci where mutations were observed in that treatment; the size of the dot corresponds to the number of mutations that occurred. Loci previously associated with compensatory mutations are highlighted in bold for all clones, and the labels for the rest of the loci were plotted only for the no-hypermutator clones to avoid overplotting. When large mutations occurred, partially deleted flanking genes appear in square brackets and completely deleted flanking genes appear in curly brackets. Download FIG S8, JPG file, 0.2 MB. Copyright © 2021 Carrilero et al. 2021 Carrilero et al. https://creativecommons.org/licenses/by/4.0/ This content is distributed under the terms of the Creative Commons Attribution 4.0 International license.

10.1128/mBio.00558-21.9 FIG S9 Growth of wild-type and compensatory mutants carrying plasmids with or without mercury. Cumulative growth was calculated as the integral of 48-h growth curves (i.e., the area under the curve; *n* = 6). Boxes show the interquartile range and the median (black bar) and mean (yellow bar). Panels show data for each of the different bacterial genotypes. Colors denote the plasmid carried by the cells: red, pQBR103; blue, pQBR57; purple, both plasmids. Color saturation and symbols denote the mercury environment: 0 μM Hg(II) chloride in darker colors and circles; 40 μM Hg(II) chloride in lighter color and triangles. Download FIG S9, JPG file, 0.3 MB. Copyright © 2021 Carrilero et al. 2021 Carrilero et al. https://creativecommons.org/licenses/by/4.0/ This content is distributed under the terms of the Creative Commons Attribution 4.0 International license.

## DISCUSSION

Plasmid coexistence is more commonly observed in bacterial genomes than is expected by chance (4), which is surprising given the high fitness costs usually associated with plasmid acquisition (1, 2, 12, 14). Here, we show that coexistence of plasmids encoding the same functional trait, namely, mercury resistance, within cells was inhibited by positive selection for this trait. With positive selection, plasmid coexistence declined due to loss of pQBR57 from originally coinfected cells, whereas without positive selection, plasmid coexistence was stably maintained. This difference could not be explained by the fitness costs of plasmid carriage, first because coexistence was maintained in environments where the net cost of plasmid carriage was highest [i.e., without Hg(II) chloride], and second because compensatory mutations to ameliorate these costs occurred across all treatments. Instead, the loss of pQBR57 from originally coinfected cells under positive selection could be explained by the differential fitness benefits provided by the two plasmids in the presence of Hg(II) chloride. Addition of Hg(II) chloride reduced the fitness of coinfected cells when competing against pQBR103 carriers but not when competing against pQBR57 carriers. Consistent with previous data (25), this occurs in spite of the pQBR103 backbone being individually costlier, because pQBR103 provides a greater fitness benefit than pQBR57 in the presence of Hg(II) chloride. Thus, where multiple plasmids encode the same functional trait, under positive selection this redundancy can drive loss of the less beneficial plasmid.

Loss of a redundant plasmid replicon by coinfected cells under positive selection is conceptually similar to the replacement of singly infected plasmid bearers by chromosomal Tn*5042*, which was also observed only under positive selection. In both cases, redundancy allows loss of one of the replicons encoding resistance. It may be the case that if our experiment had been run for even longer, the initially coinfected populations would also eventually consist only of cells with chromosomal Tn*5042*. Indeed, our relative fitness data show that SBW25(Tn*5042*) outcompeted both singly infected and coinfected plasmid bearers in mercury-containing environments. This outcome may have been delayed (or prevented) because the loss of both plasmids by segregation is likely to occur with low probability and/or because the fitness difference between chromosomal and plasmid-encoded mercury resistance was reduced by compensatory evolution. The key difference between environments with and those without positive selection is that in the former, both the benefit and the cost of the plasmids contribute to their fitness effect, whereas in the latter only their costs do. Accounting for the beneficial effects of plasmid carriage under positive selection appears to increase the fitness difference between coinfected cells and those carrying only the more beneficial plasmid. Selection for plasmid benefits may therefore be more efficient than negative selection against plasmid costs, which becomes less efficient over time due to compensatory evolution to ameliorate the plasmid fitness cost.

Previous studies suggest that the positive epistasis of plasmid fitness costs enables plasmid coexistence in bacterial genomes (4). Our data provide contradictory evidence both for and against this idea. When competing against a plasmid-free genotype [SBW25(Tn*5042*)], SBW25 coinfected with both plasmids showed a lower fitness cost than expected from the individual fitness costs of each plasmid alone. This is consistent with positive epistasis. However, when coinfected SBW25 was directly competed against SBW25 singly infected with either of the plasmids, we measured an appreciable additional cost of plasmid coinfection. This is not consistent with positive epistasis. It seems likely that direct competition of coinfected versus singly infected cells offers the most accurate method to measure the fitness effect of carrying a second plasmid replicon, whereas competing singly infected and coinfected cells separately against a plasmid-free competitor seems likely to underestimate the actual cost of carrying a second plasmid. Importantly, the study by San Millan and colleagues used a setup similar to our first set of experiments, namely, separate competition against a common plasmid-free strain (4), a method that our data suggest is likely to underestimate the actual fitness cost of being coinfected. Therefore, taken together, our data do not support a role for positive epistasis in explaining plasmid coinfection and they suggest that caution should be exercised when epistasis from relative fitness data obtained against plasmid-free competitors is being interpreted.

At present, it is unclear why the two plasmids differ in the benefit they provide to SBW25 under positive mercury selection. Both plasmids possess one copy of the Tn*5042* transposon encoding the Mer operon, which provides mercury resistance. These Tn*5042* sequences are identical except for a single base pair difference in *merR*, a repressor controlling expression of the Mer operon (25). Low concentrations of Hg(II) are bound by MerR, causing a conformational change of the protein which relieves repression, allowing expression of the Mer operon, which imports Hg(II) into the cell and reduces it to Hg (32, 33). It is possible that the single base pair difference in the *merR* sequence between the pQBR103 and pQBR57 copies of Tn*5042* could change the sensitivity of the MerR repressor, leading to altered expression of the Mer operon. For example, by detoxifying the environment through reduction of Hg(II) to Hg, mercury resistance benefits all neighboring cells (34); thus, a reduced affinity for Hg(II) could accrue fitness benefits during competition with strains encoding MerR with a higher affinity and thus earlier expression of the Mer operon. Alternatively, the plasmids differ extensively in their gene content, and it is possible that epistatic interactions between these variable genes and the Mer operon could underlie the observed differential fitness benefits. Resolving this is beyond the scope of this paper, but identifying the causes of differences in plasmid benefit will be a focus of future work.

We observed mutations in three loci previously associated with compensatory evolution for pQBR plasmids. Specifically, mutations to either of the genes encoding the GacAS two-component global regulatory system (18, 19) or to *PFLU4242*, encoding a protein of unknown function (18), were sufficient to ameliorate the fitness costs associated with being coinfected by both pQBR103 and pQBR57. Similarly, deletion of either locus could completely ameliorate the cost of pQBR103, whereas amelioration of pQBR57 was more complete via deletion of *PFLU4242* than *gacS*. Together, these observations suggest that these plasmids, despite being genetically divergent, cause their associated fitness costs through a similar (or a shared) mechanism. Shared targets of compensatory evolution among divergent plasmids have been reported in other plasmid-host systems (16, 20), for example, compensatory mutations in chromosomal helicases (20). Consistent with previous work on other host-plasmid systems (16, 20), our findings suggest that compensatory evolution could indeed promote plasmid coexistence by reducing the cost of multiple plasmids simultaneously. This has the effect of reducing the efficiency of negative selection against plasmid costs over time. It is notable, however, that although compensatory evolution ameliorating the fitness costs of being coinfected occurred in both the mercury-containing and mercury-free environments, plasmid coexistence was stable only in the mercury-free environment (at least for the duration of this study). This occurs because positive selection remains efficient even after compensatory evolution occurs, allowing discrimination of differential fitness benefits among functionally redundant plasmid replicons.

Although fitness differences do appear to explain the patterns we observed, it is possible that additional factors not considered here also contributed to the contrasting plasmid dynamics in the presence versus absence of positive selection. Addition of mercury could have altered the segregation or conjugation rates of the plasmids, for instance, by disrupting the formation of disulfide bonds in proteins involved in these processes prior to reduction of Hg(II) ions to Hg by MerA (35). If pQBR57 was impacted more strongly than pQBR103 by the addition of mercury, for example, by suffering an increased segregation rate or reduced conjugation rate, this could work together with the differential fitness costs and benefits of the plasmids we measured to accelerate the loss of pQBR57 from coinfected cells in Hg(II)-containing environments. These possibilities remain to be tested in future work.

These data reveal that multiple plasmids are unlikely to be maintained in a genome under positive selection if they encode redundant functions and there are differences in the fitness benefits these accessory genes provide. Moreover, while compensatory evolution can promote plasmid coexistence in the absence of positive selection, it does not do so in the presence of positive selection, where not only the cost but also the benefit of the plasmid is subject to selection. These findings suggest, therefore, that widespread plasmid coexistence in bacterial genomes is likely to be explained either by positive selection for accessory gene functions being rare in nature or by coinfecting plasmids encoding nonredundant accessory gene functions. These findings have implications for understanding the forces structuring bacterial genomes (1, 5–7) and suggest a process whereby recurrent phases of genome expansion and contraction are driven by variable positive selection: multiple redundant replicons can be acquired, and their costs ameliorated by compensatory evolution, thus allowing genome expansion between bouts of periodic positive selection, which, when they occur, select reduced genomes containing only the highest-benefit replicon(s). This process is likely to be reinforced by the copying and pasting of transposons encoding functional genes between plasmids (10), creating further redundancy among coexisting replicons.

## MATERIALS AND METHODS

### Bacterial strains and culture conditions.

Bacterial populations were grown in liquid King’s B (KB) (36) broth microcosms (6 ml of KB broth in a 30-ml glass universal vial). These were incubated at 28°C and shaken at 180 rpm. To generate positive selection for mercury resistance, microcosms were supplemented with 40 μM Hg(II) chloride, as required. Bacterial colonies were obtained by plating serial dilutions onto KB agar. To select particular bacterial strains (described below), agar plates were supplemented with gentamicin (30 μg/ml), streptomycin (250 μg/ml), Hg(II) chloride (20 μM for plating and 100 μM for replica plating), kanamycin (25 μg/ml), or X-Gal (5-bromo-4-chloro-3-indolyl-β-d-galactopyranoside; 75 μg/ml), as required.

Two isogenic P. fluorescens SBW25 strains (23) with chromosomal resistance markers (either gentamicin resistance [Gm^r^] or streptomycin resistance with LacZ [Sm^r^*lacZ*]) were used to enable creation of transconjugants (25, 30, 37). Derived P. fluorescens SBW25 mutants with deletion of *gacS* (SBW25-Gm^r^-ΔgacS) (19) or *PFLU4242* (SBW25-Gm^r^-ΔPFLU4242) (18) were used to enable measurement of the effects of these genes on plasmid costs in competition experiments. An isogenic plasmid-free P. fluorescens SBW25 Gm^r^ strain with a chromosomal Tn*5042* mercury resistance transposon derived from the pQBR103 plasmid [SBW25(Tn*5042*)] was used to enable measurement of the fitness cost of the plasmid backbones in competition experiments (29).

Two mercury resistance plasmids were used in this study that had been previously isolated from agricultural soil in Oxfordshire, United Kingdom: pQBR57 (23) and pQBR103 (24). An isogenic variant of pQBR103 marked with an mCherry fluorescent protein gene and a kanamycin resistance gene (mCherryKm^r^) was used to enable selection of coinfected bacterial cells (31). Plasmids were introduced to bacterial strains by conjugation using standard protocols (25, 38). Briefly, transconjugants were selected by plating onto KB agar plates supplemented with 20 μM Hg(II) chloride or 25 μg/ml kanamycin and the relevant antibiotic (either gentamicin [30 μg/ml] or streptomycin [250 μg/ml]) as appropriate to select the recipient strain. Plasmid status of transconjugant colonies was determined by PCR, as previously described (19, 28).

### Selection experiment.

Six replicate populations each of SBW25-Sm^r^*lacZ*(pQBR57+pQBR103-Km^r^), SBW25-Sm^r^*lacZ*(pQBR57), and SBW25-Sm^r^*lacZ*(pQBR103-Km^r^) were propagated with or without positive selection [i.e., supplementation with 40 μM Hg(II) chloride or 0 μM Hg(II) chloride, respectively], and six replicate populations of the SBW25-Sm^r^*lacZ* plasmid-free control were propagated without Hg(II) chloride. [Note that plasmid-free populations cannot survive in 40 μM Hg(II) chloride.] Each replicate was founded by 60 μl of an overnight liquid culture initiated from a single independent colony previously streaked on KB agar. One percent of each population was serially transferred to fresh medium every 48 h for 40 transfers, resulting in approximately 265 bacterial generations. Every 10 transfers, serial dilutions of each population were plated onto KB agar and incubated at 28°C to enumerate bacterial densities. These plates were replica plated onto KB agar supplemented with Hg(II) chloride at 100 μM to determine the frequency of mercury resistance (Hg^r^). Twenty-four mercury-resistant colonies per population per time point were chosen at random to determine the presence of each plasmid and Tn*5042* by multiplex PCR. We used three set of primers to target the Mer-Tn*5042* transposon (forward, TGCAAGACACCCCCTATTGGAC; reverse, TTCGGCGACCAGCTTGATGAAC), the pQBR103-plasmid specific origin of replication *oriV* (forward, TGCCTAATCGTGTGTAATGTC; reverse, ACTCTGGCCTGCAAGTTTC) and the pQBR57-plasmid specific *uvrD* gene (forward, CTTCGAAGCACACCTGATG; reverse, TGAAGGTATTGGCTGAAAGG) (19, 28). Briefly, a mixture of 1× GoTaq Green (Promega, WI, USA) with a 0.71 μM concentration of each primer to detect pQBR103, a 0.89 μM concentration of each primer to detect pQBR57, and a 0.36 μM concentration of each primer to detect Tn*5042* was used with the following thermocycling program: 95°C for 5 min; 30 cycles of 95°C for 30 min, 58°C for 30 min, and 72°C for 1 min; and 72°C for 5 min (28).

### Competition experiments.

Competition experiments were used to measure the fitness costs associated with carrying a plasmid(s) against a range of competitor strains. In all cases, overnight cultures of competitors were mixed in a 1:1 ratio, diluted 100-fold into KB microcosms with and without 40 μM Hg(II) chloride, and incubated for 48 h at 28°C with shaking at 180 rpm. Starting and final densities of each marked strain were determined by plating onto KB agar supplemented with X-Gal, and relative fitness (*w*) was calculated as previously described (19).

To measure the fitness cost of the plasmid backbones *per se*, that is, the cost once the fitness effect of the Tn*5042* is accounted for, the plasmid-bearing strains [SBW25-Sm^r^*lacZ*(pQBR103-Km^r^), SBW25-Sm^r^*lacZ*(pQBR57), and SBW25-Sm^r^*lacZ*(pQBR57+pQBR103-Km^r^)] were competed against SBW25-Gm^r^(Tn*5042*) both with and without Hg(II) chloride. Six replicates were performed per comparison.

To measure the fitness cost of plasmid coinfection, we competed SBW25-Sm^r^*lacZ*(pQBR57+pQBR103-Km^r^) against either SBW25-Gm^r^(pQBR103-Km^r^), or SBW25-Gm^r^(pQBR57), or SBW25-Gm^r^(pQBR57+pQBR103-Km^r^) as a control, both with and without Hg(II) chloride. Twelve replicates were performed per comparison. Normalized fitness was calculated by subtracting the mean of the control competition [i.e., SBW25-Sm^r^LacZ(pQBR57+pQBR103-Km^r^) versus SBW25-Gm^r^(pQBR57+pQBR103-Km^r^)] to account for any difference in fitness attributable to the selectable markers used.

To measure the fitness effect of putative compensatory mutations on plasmid carriage, we competed SBW25-Gm^r^, SBW25-Gm^r^-ΔgacS, or SBW25-Gm^r^-ΔPFLU4242 carrying a plasmid(s) (pQBR57, pQBR103-Km^r^, or pQBR57+pQBR103-Km^r^) against plasmid-free SBW25-Sm^r^*lacZ*. Six replicates were performed per comparison.

### Genomic analysis.

The whole-genome sequence for at least one randomly chosen clone per population was obtained at the end of the selection experiment. For populations containing multiple mercury-resistant genotype subpopulations—i.e., clones without mercury resistance or with Tn*5042* only, pQBR103 only, pQBR57 only, or both pQBR57 and pQBR103—and where these genotypes comprised at least 10% of the population, we obtained the whole-genome sequence for one randomly chosen clone per subpopulation. Whole-genome sequencing was performed by MicrobesNG using a 250-bp paired-end protocol on the Illumina HiSeq platform. Paired reads were aligned to the annotated ancestral genome sequence using the Burrows-Wheeler Aligner (39), and duplicate reads were removed using picard (https://broadinstitute.github.io/picard/). Variants were called using GATK Haplotype Caller (40) and annotated using SnpEff (41). Called variants were then filtered to remove low-quality calls with either low coverage (<12 reads per bp), low quality (scores <200), or low frequency of the alternative allele (<90% of reads with alternative). In addition, as a complementary and confirmatory approach, variants were also called against the ancestral reference genome using the Breseq computational pipeline with the standard default settings (42). All variants not called by both methods were validated visually using the alignment viewer IGV (43, 44).

### Statistical analysis.

The integral of mercury resistance frequency over time was calculated as the area under the curve using the AUC function of the flux package in R and compared between treatments using Welch’s ANOVA due to unequal variances. *Post hoc* pairwise comparisons were performed using the Dunnett T3 test. The integral of plasmid coinfection frequency over time was calculated as the area under the curve using the AUC function of the flux package in R and compared between treatments using a Mann-Whitney U test due to unequal variances. Relative fitness data from competition experiments was analyzed using ANOVA, and *post hoc* pairwise comparisons were performed using Tukey tests. Analyses were performed in R 3.6.1 (45) or Prism v8.1.2.

### Data availability.

All sequencing data are available in the Short Read Archive under accession numbers PRJEB38218 and ERP121615.
