# Supplementary figures and images for: Positive Selection Inhibits Plasmid Coexistence in Bacterial Genomes
Source: mBio. 2021 May 11;12(3):e00558-21. doi: 10.1128/mBio.00558-21 (PMC8262885; doi:10.1128/mBio.00558-21)

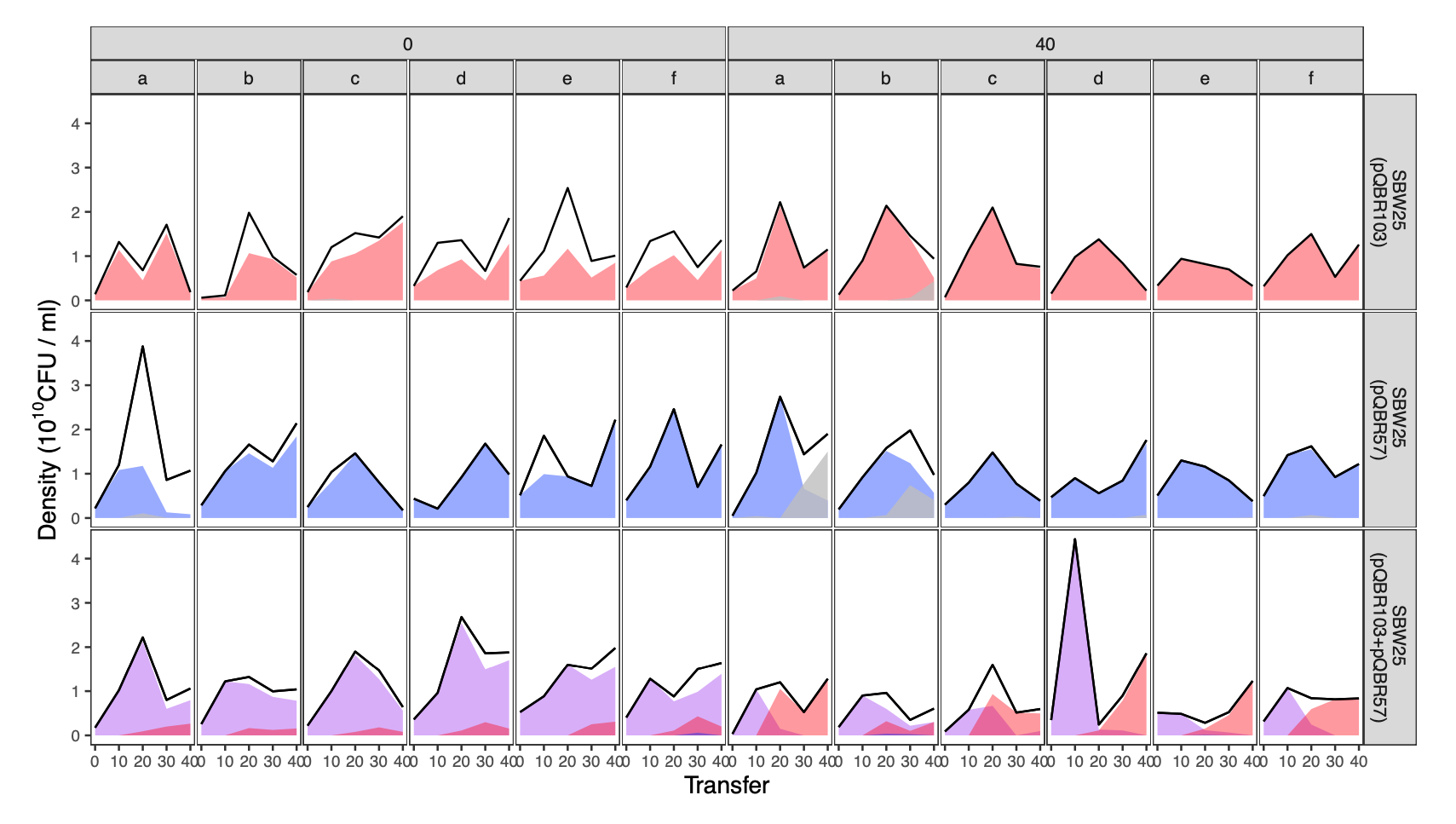

Supplement: FIG S1 [file mbio.00558-21-sf001.jpg]

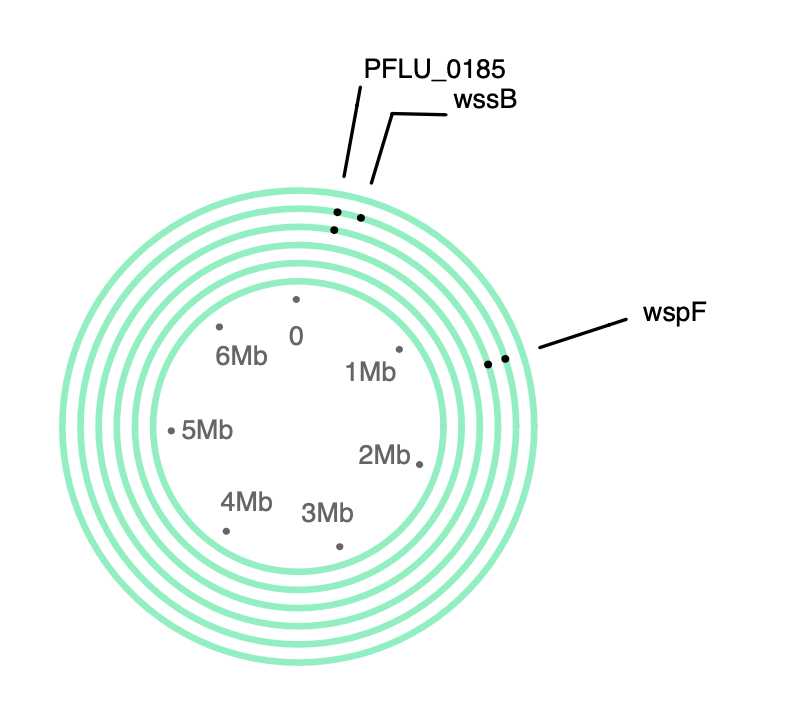

Supplement: FIG S2 [file mbio.00558-21-sf002.jpg]

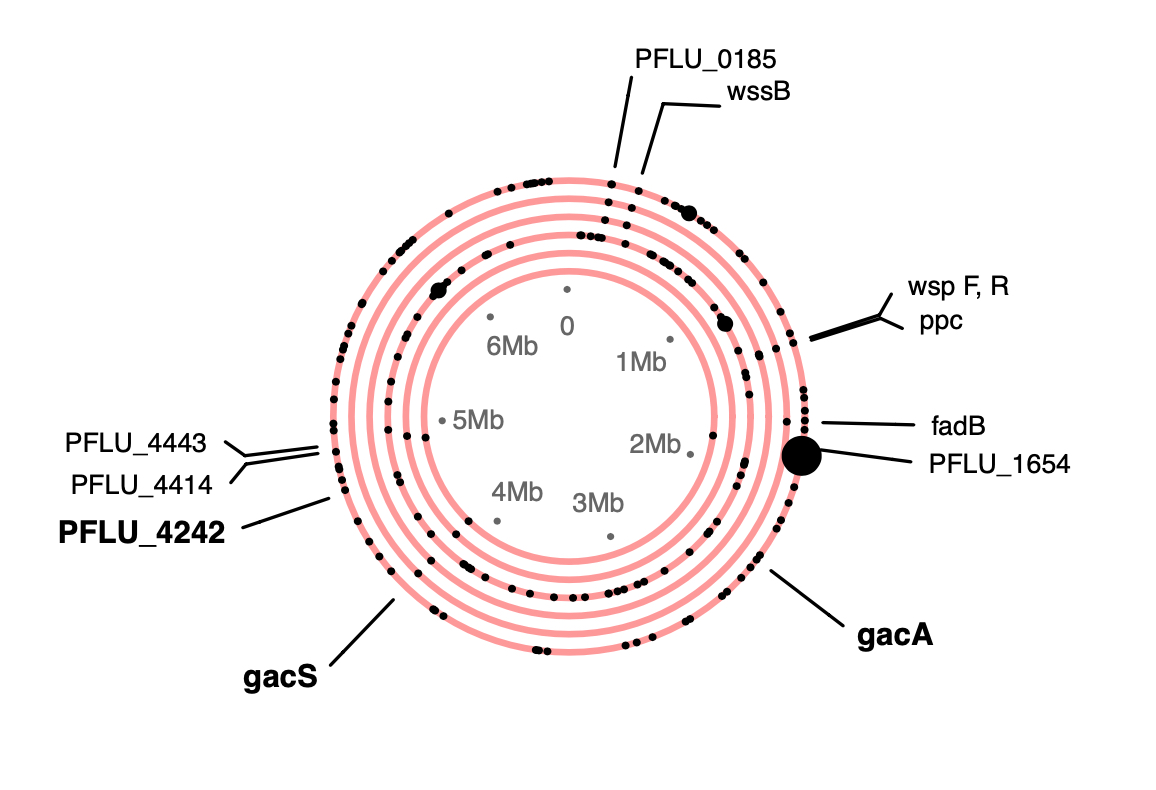

Supplement: FIG S3 [file mbio.00558-21-sf003.jpg]

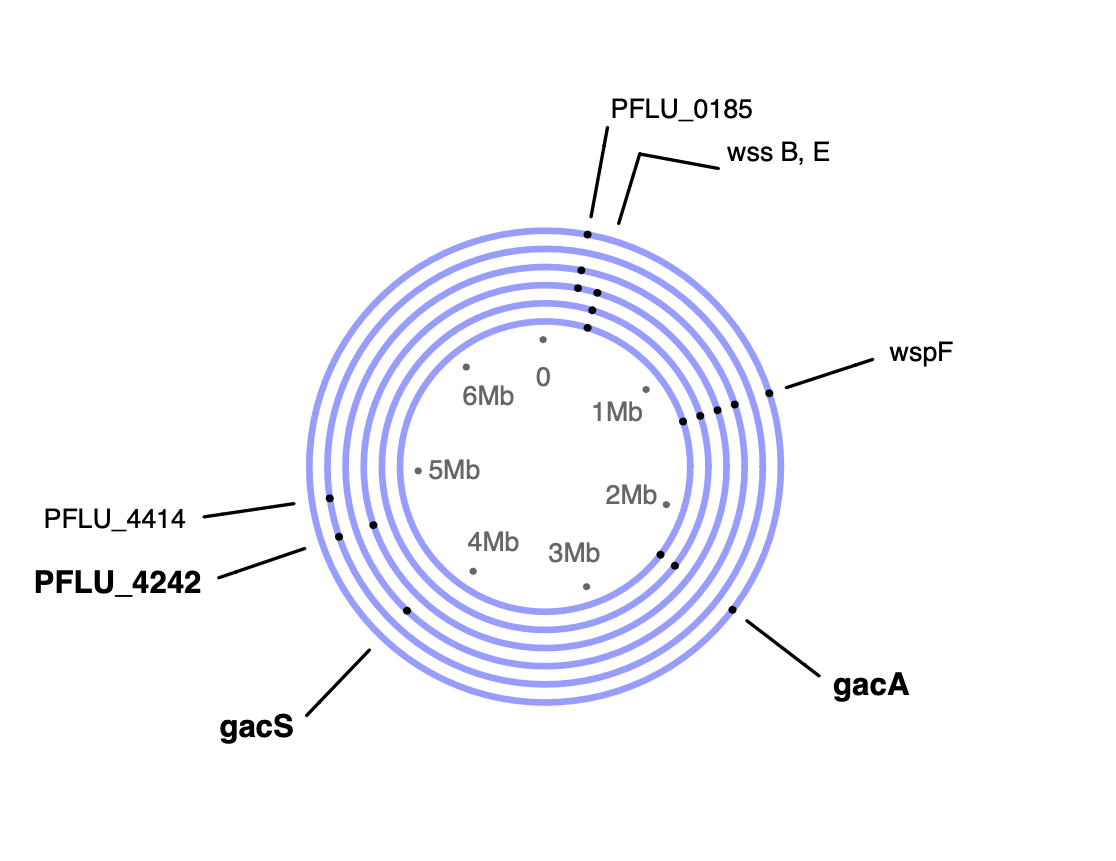

Supplement: FIG S4 [file mbio.00558-21-sf004.jpg]

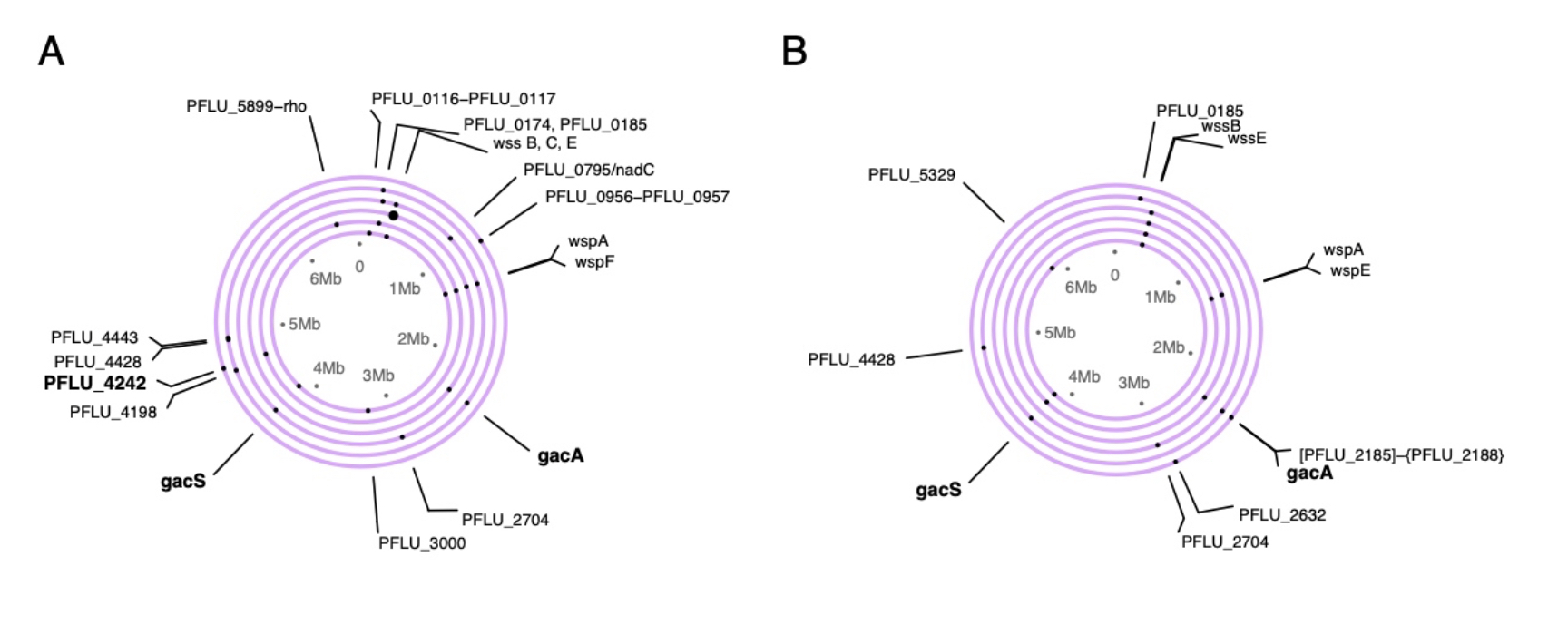

Supplement: FIG S5 [file mbio.00558-21-sf005.jpg]

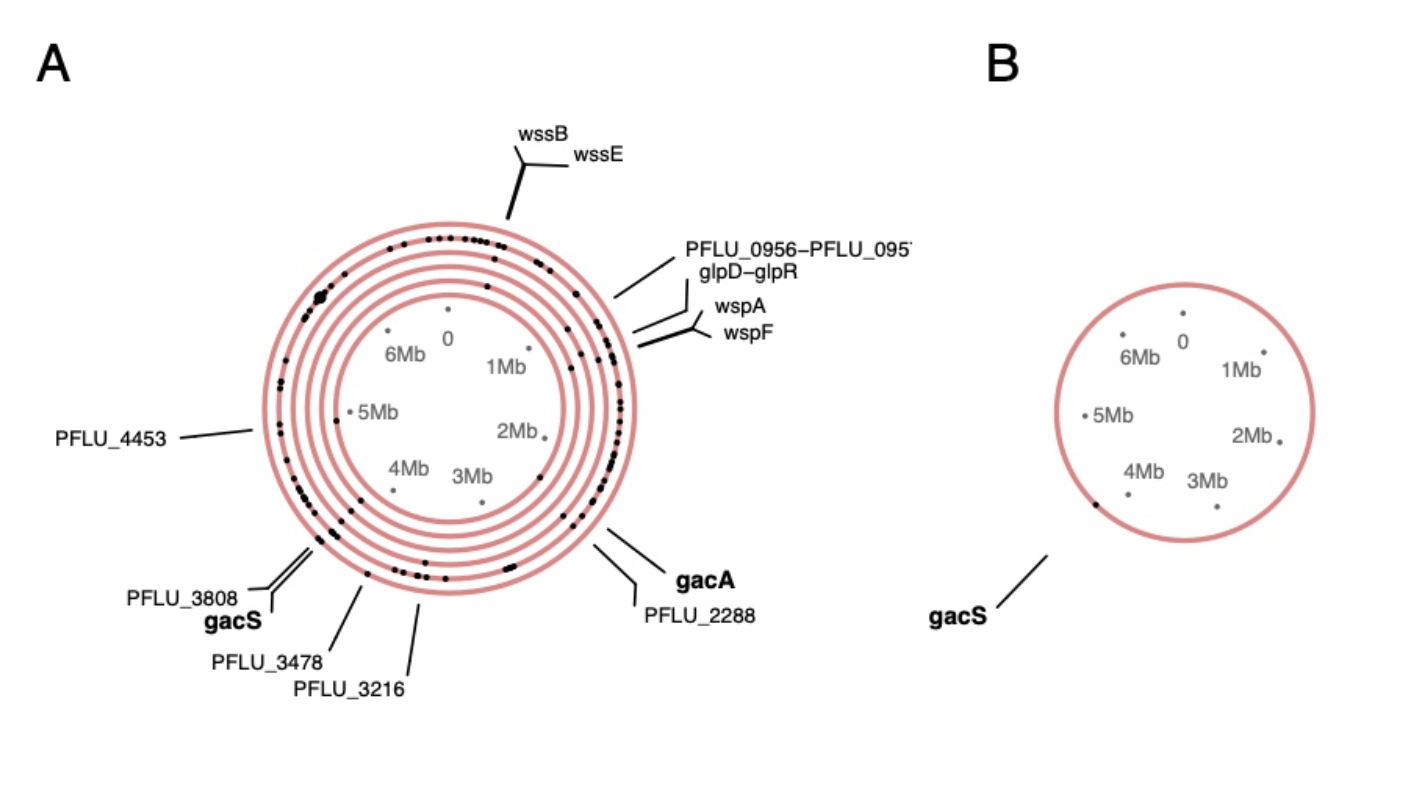

Supplement: FIG S6 [file mbio.00558-21-sf006.jpg]

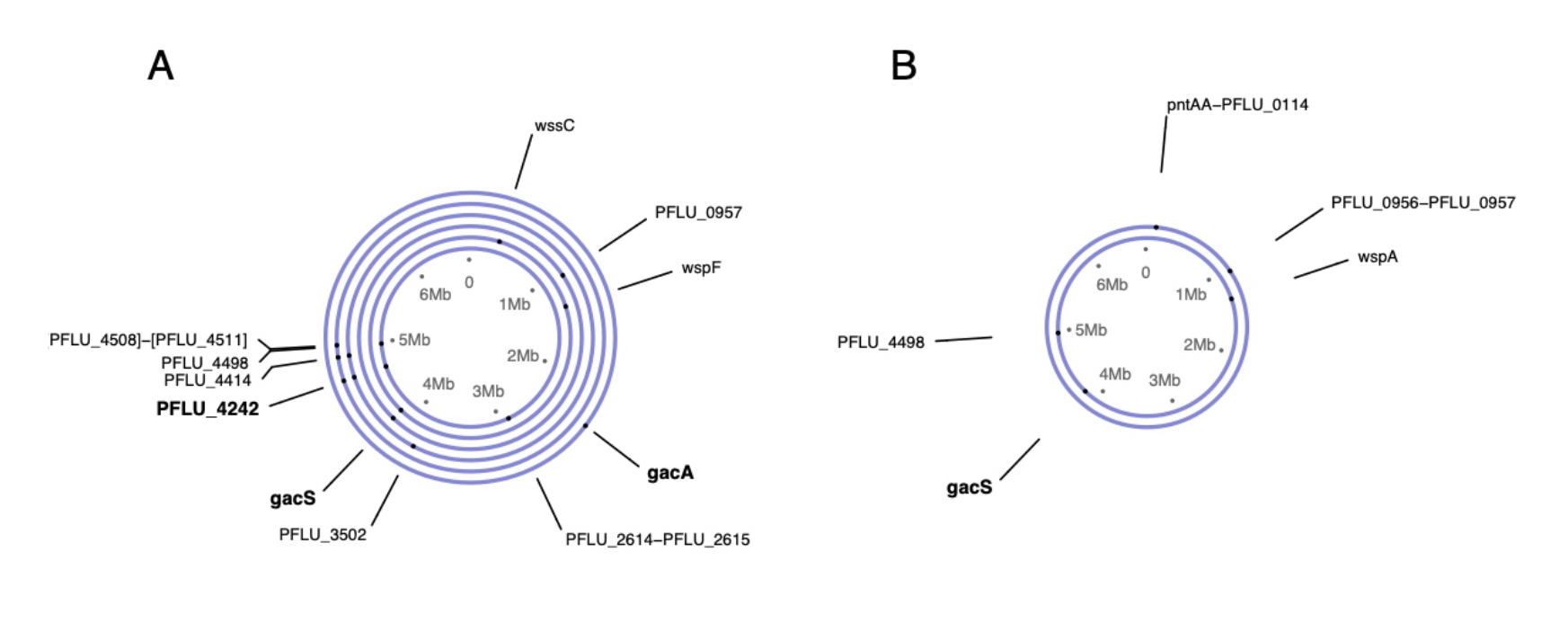

Supplement: FIG S7 [file mbio.00558-21-sf007.jpg]

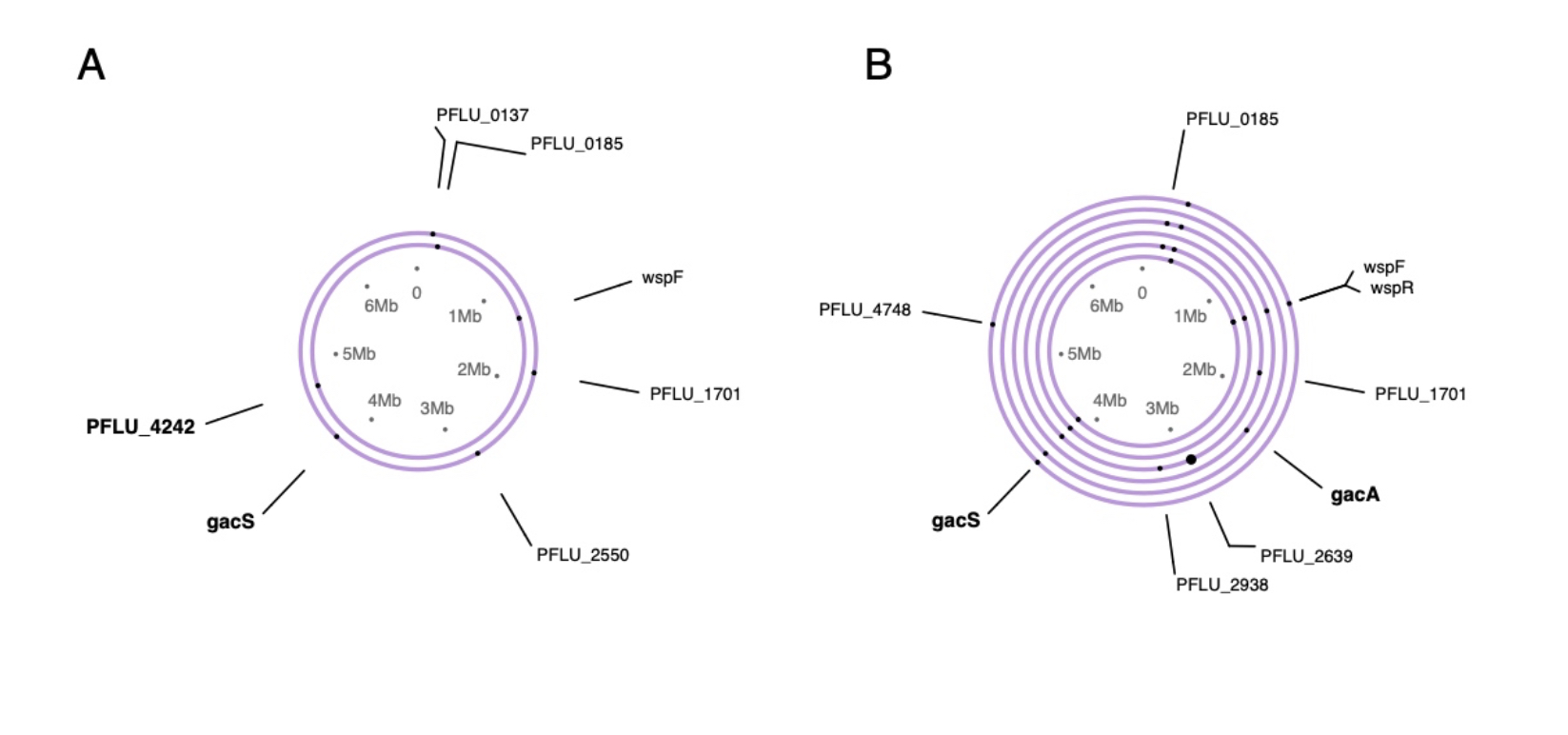

Supplement: FIG S8 [file mbio.00558-21-sf008.jpg]

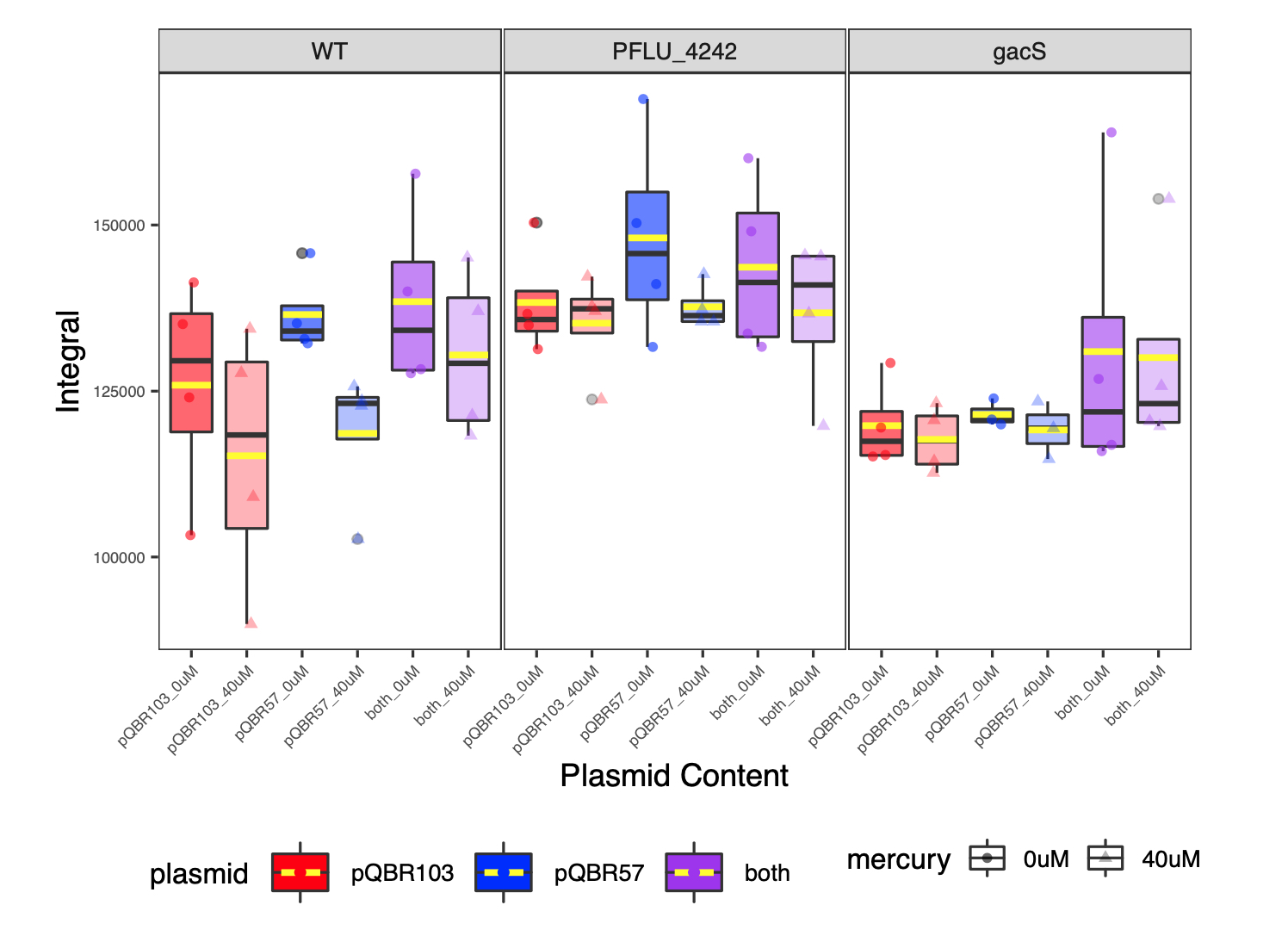

Supplement: FIG S9 [file mbio.00558-21-sf009.jpg]
